# A convolutional neural network model for EPID‐based non‐transit dosimetry

**DOI:** 10.1002/acm2.13923

**Published:** 2023-03-02

**Authors:** Lucas Dal Bosco, Xavier Franceries, Blandine Romain, François Smekens, François Husson, Marie‐Véronique Le Lann

**Affiliations:** ^1^ Laboratoire d'Analyse et d'Architecture des Systèmes (LAAS) Toulouse France; ^2^ Institut National de la Santé Et de la Recherche Médicale (INSERM) Toulouse France; ^3^ Department of Physics DOSIsoft SA Cachan France

**Keywords:** deep‐learning, EPID‐based dosimetry, gamma‐index analysis, pre‐treatment verification, U‐net

## Abstract

**Purpose:**

To develop an alternative computational approach for EPID‐based non‐transit dosimetry using a convolutional neural network model.

**Method:**

A U‐net followed by a non‐trainable layer named True Dose Modulation recovering the spatialized information was developed. The model was trained on 186 Intensity‐Modulated Radiation Therapy Step & Shot beams from 36 treatment plans of different tumor locations to convert grayscale portal images into planar absolute dose distributions. Input data were acquired from an amorphous‐Silicon Electronic Portal Image Device and a 6 MV X‐ray beam. Ground truths were computed from a conventional kernel‐based dose algorithm. The model was trained by a two‐step learning process and validated through a five‐fold cross‐validation procedure with sets of training and validation of 80% and 20%, respectively. A study regarding the dependance of the amount of training data was conducted. The performance of the model was evaluated from a quantitative analysis based the ϒ‐index, absolute and relative errors computed between the inferred dose distributions and ground truths for six square and 29 clinical beams from seven treatment plans. These results were also compared to those of an existing portal image‐to‐dose conversion algorithm.

**Results:**

For the clinical beams, averages of ϒ‐index and ϒ‐passing rate (2%‐2mm > 10% D_max_) of 0.24 (±0.04) and 99.29 (±0.70)% were obtained. For the same metrics and criteria, averages of 0.31 (±0.16) and 98.83 (±2.40)% were obtained with the six square beams. Overall, the developed model performed better than the existing analytical method. The study also showed that sufficient model accuracy can be achieved with the amount of training samples used.

**Conclusion:**

A deep learning‐based model was developed to convert portal images into absolute dose distributions. The accuracy obtained shows that this method has great potential for EPID‐based non‐transit dosimetry.

## 1 INTRODUCTION

Over the past 20 years, external beam radiotherapy has become more complex with the development of increasingly accurate treatment techniques. For instance, Intensity‐Modulated Radiation Therapy (IMRT) aims to improve tumor target coverage with the extensive use of the Multi Leaf Collimator (MLC), while Volumetric Modulated Arc Therapy adds a degree of modulation through dose rate variation and continuous gantry rotation. To meet regulatory requirements for quality and safety in clinical routine, these techniques require additional attention.^1^ As part of patient‐specific Quality Assurance (QA), pre‐treatment verification guarantees that the beam fluence initially planned by the Treatment Planning System (TPS) can be correctly delivered by the linear accelerator (LINAC). More specifically, the objective of this check is to detect, prior to each first patient treatment fraction, possible data integrity issues, beam output variations or mechanical errors of collimation elements.^2^

The amorphous‐Silicon (a‐Si) Electronic Portal Image Device (EPID) is a planar digital detector taking into account the beam fluence. The EPID has a good reproducibility, a high signal‐to‐noise ratio, provides high‐resolution 2D digital images and its response is considered linear with the dose.^3^, ^4^ These different qualities make it an ideal tool for QA purposes such as non‐transit dosimetry, that is, acquisitions without attenuator between the radiation source and the imager. However, its use for pre‐treatment verification requires dosimetric bias correction and absolute dose calibration.^5^

**FIGURE 1:**
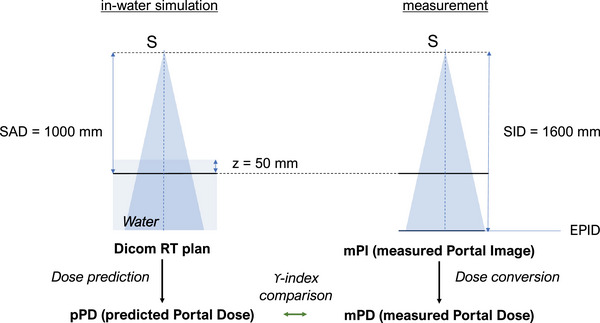
Reference geometry of the direct method for pre‐treatment verification. SAD (Source‐Axis Distance), SID (Source‐Imager Distance).

As a result, two approaches for non‐transit dosimetry exploiting the assets offered by EPID have emerged. First, the back‐projection method which involves extracting the primary fluence from the raw EPID signal to perform a TPS‐like dose calculation in the patient's planned computed tomography.^6^, ^7^ The resulting dose distribution is compared to the planned dose distribution using the gamma‐index (γ‐index) metric.^8^ Second, the direct method displayed in Figure 1, which is based on the comparison between the measured Portal Dose distribution (mPD) and the predicted Portal Dose distribution (pPD).^9^ The pPD is simulated in a virtual water phantom at a given depth from the data of the DICOM RT plan file, while the mPD is computed from the measured Portal Image (mPI) with an EPID grayscale to dose‐to‐water conversion algorithm. For this purpose, models based on measurements^10^, ^11^ or kernels^12^, ^13^, ^14^, ^15^ have been proposed. The latter offer sufficient accuracy for delivery error detection^9^, ^16^ despite the approximation of physics modeling required for their commissioning.

Recently, the rise of Machine Learning (ML) has enabled the development of many applications for which it was previously difficult to find reliable solutions with current parametric models.^17^ In the field of radiotherapy, the contribution of ML to QA is major. For instance, ML was experimented for the automation of QA processes^18^, ^19^, ^20^, ^21^ and a new approach to current analytical algorithms to compute dose distributions for EPID‐based non‐transit dosimetry.^22^

Regarding the latter, three studies experimented with the use of artificial neural networks (ANNs) to convert mPIs into planar mPDs for pre‐treatment verification of IMRT beams. Khalantzis et al.^23^ proposed the extraction of two clusters in the fluence domain using a K‐means algorithm to train two MultiLayer Perceptrons (MLPs) dedicated to high and low dose regions, respectively. Mahdavi et al.^24^ developed a single MLP to compute mPDs from the raw EPID signal. Chatrie et al.^25^ extended a similar approach to other EPID models and tumor sites. The results obtained by these previous studies were encouraging. However, the use of MLPs for image regression tasks has drawbacks. For instance, if entire images are used as input, each input neuron is dedicated to a single pixel, resulting in excessive memory usage. Patches can be used but an assumption about the appropriate size is made. In addition, this inherently restricts the receptive field of the model. Finally, it was shown that the densely‐connected layer connections are redundant which complicates learning for image processing tasks.^26^

With significant success in the field of Deep Learning (DL), Convolutional Neural Networks (CNNs) are a type of ANNs specifically designed to process data in the form of sequences or images.^26^ In particular, the U‐net originally proposed by Ronnerberg et al. in 2015,^27^ has become the reference CNN architecture for image regression applications. More specifically, this model is a fully‐convolution network composed of an encoder, a bottleneck and a decoder forming a single structure. In the field of medical physics, the U‐net has been tested for dose and dose rate computation,^28^, ^29^, ^30^, ^31^, ^32^ denoising of CT images,^33^ correction of mPIs acquired from MR‐LINAC^34^ and conversion of MR signal to density matrix.^35^

Compared to MLP, the use of U‐net for the mPI‐to‐mPD conversion should be more suitable. Indeed, the use of convolutional layers and dimensionality reduction provide more efficient features extraction while optimizing the number of trainable parameters.^26^ This reduces memory usage and increases learning efficiency. In addition, compared to MLP neurons, the convolution kernels slide through features maps, enabling CNNs to process an entire image of any size in a single forward pass. However, the use of CNNs can result in insufficient encoding of spatialized information.^36^ This can be an obstacle for mPI‐to‐mPD conversion where pixel‐wise transformations, such as beam profile restoration,^37^ are required.

In this study, we investigate the use of CNNs for the conversion of grayscale mPIs to absolute mPDs for pre‐treatment verification of IMRT Step & Shoot (S&S) beams of various tumor locations. The proposed model is based on an adapted U‐net architecture. A set of preprocessed mPIs were directly used as input data and the reference pPDs computed by a conventional kernel‐based dose algorithm were used as output data. A non‐trainable layer called True Dose Modulation (TDM) combined with a two‐step learning process were also introduced to efficiently recover the spatialized information.

In the first section of this paper, the equipment used, the data acquisition, and the database distribution are described. The True Dose Modulation layer, the proposed U‐net architecture, the training process as well as the model evaluation are also presented. In the second section, results on elementary and clinical cases are described, compared to existing methods, and discussed.

## 2 METHODS

### 2.1 Database

All mPIs were acquired with the synergy LINAC (Elekta, Stockholm, Sweden) equipped of an Agility MLC of 80 leaf‐pairs with a 6 MV X‐ray beam and a nominal dose rate of 400 MU/min. The a‐Si flat panel EPID iView GT (Perkin Elmer Optoelectronics, Wiesbaden, Germany) was used for mPI acquisitions in integrated mode. This EPID model is positioned at a source‐imager distance of 1600 mm and is provided with a 1024 × 1024 pixel array equivalent to 24.5 × 24.5 cm^2^ active area at source‐axis distance (SAD), yielding a pixel pitch of 0.24 mm. In this study, the EPID was centered on the beam axis. All acquisitions were achieved within 1‐month interval and without additional build‐up material. Acquired mPIs are 16‐bits grayscale encoded and stored with DarkField and FloodField corrections.

The reference pPDs correspond to the 2D absolute dose distributions located at SAD, at 50 mm depth in a virtual water phantom. The computation of pPDs was performed by the prediction algorithm of the EPIbeam system (version 1.05, DOSIsoft, Cachan, France). This software uses data from the DICOM RT plan file, and a kernel‐based dose engine parametrized from dose distributions obtained by Collapsed Cone Convolution with the RayStation TPS (version 1.08, RaySearch Laboratories, Stockholm, Sweden). To compare the developed model with existing methods, the EPIbeam system was also used to compute mPDs from its EPID grayscale‐to‐dose conversion algorithm. The latter is parametrized from the prediction algorithm used for the pPD computations.

The data reliability was ensured by a preliminary verification. For this purpose, each sample was visually validated by a medical physicist and quantitatively assessed through the computation of the global ϒ‐index between pPDs and mPDs with 2%‐2mm criteria and a minimum threshold at 10% of the maximum reference dose (2%‐2 mm > 10% D_max_). In this manner, the absence of beam output variations or mechanical errors of collimation elements was guaranteed. For the entire database, obtained ϒ‐passing rates were greater than 95%.

In this study, a training database consisting of 186 samples (mPI‐pPD pairs) from 36 IMRT S&S treatment plans was used for a cross‐validation procedure (see Section 2.4). Twenty nine IMRT S&S beams from seven treatment plans, excluded from the training set, and six square beams exposed to 100 MU with 15 mm, 50 mm, 80 mm, 100 mm, 150 mm, and 200 mm side were used for the evaluation of the selected model. More details about the data used in this study are available in the Tables 2 and 3 in the Appendix A.

**FIGURE 2:**
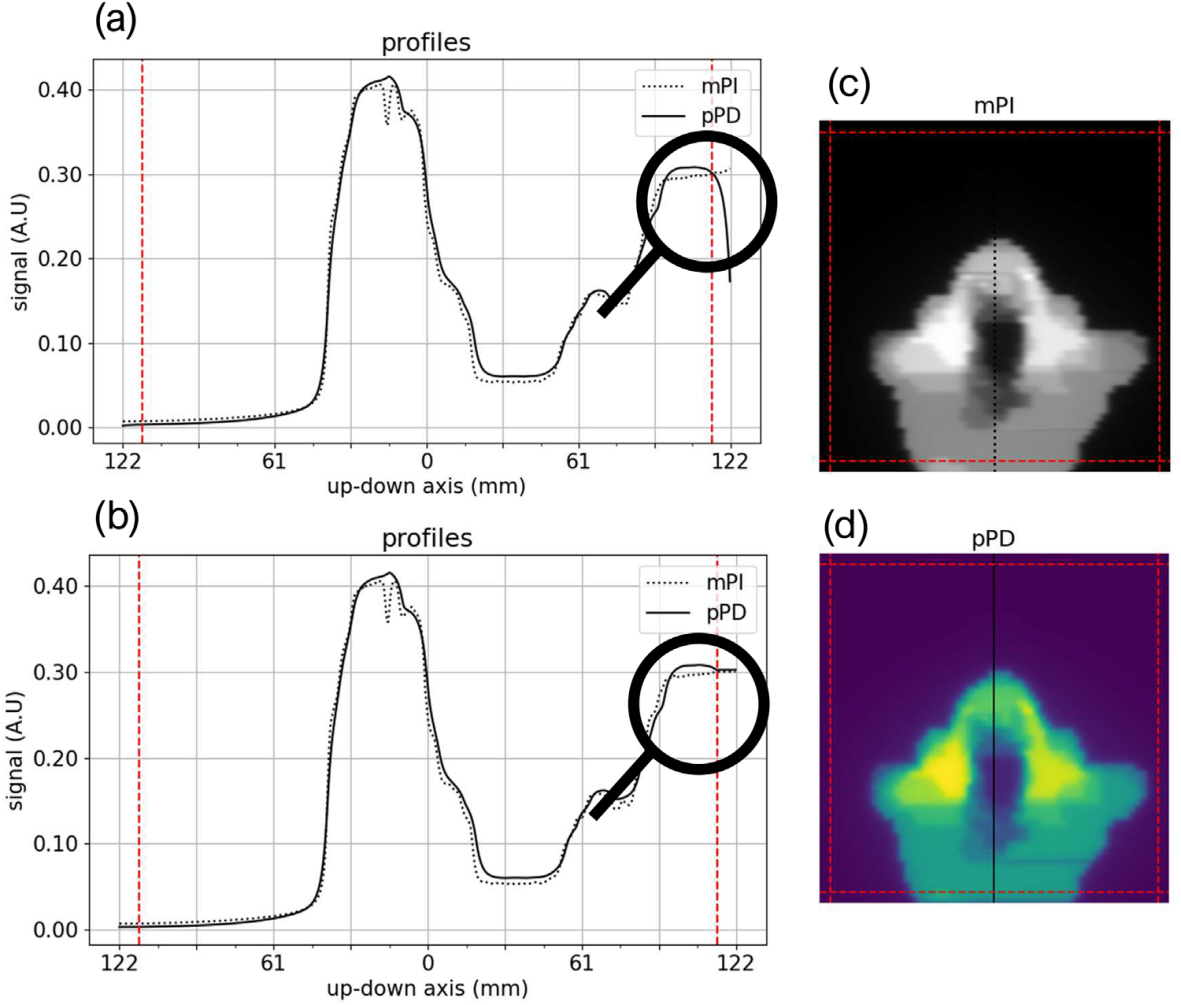
mPI (c), pPD (d) and their up‐down profiles centered on the beam axis before (a) and after (b) the edge correction for a Head & Neck IMRT S&S training beam. On each side of the profiles and images, the dashed red line represents the crop boundary applied on mPIs and pPDs.

Prior to the learning phase, preprocessing of the training data was performed. First, to ensure consistency between input and output data, the mPIs and pPDs were cropped with 32 pixels on each side and extrapolated by nearest neighbors. This edge correction is applied because an erosion is made by the analytical prediction model on pPDs for beams protruding from the EPID surface (see Figure 2). All data were also rescaled to ensure model convergence and better performance. The input and output normalization factors were, respectively, defined as the central pixel value of the mPI and pPD of the 80 mm square beam. During inferences, the output normalization factor (Gy) was also used for absolute dose calibration of inferred mPDs.

### 2.2 True dose modulation layer

Although CNNs are known for their spatial invariance,^38^ it was shown that convolutional layers are able to encode spatialized information by exploiting image boundaries, in particular using large receptive fields and zero‐padding.^39^, ^40^ However, when tasks explicitly depend on the absolute position of pixels, the recovery of spatialized information may not be sufficiently accurate.^36^ For mPI‐to‐mPD conversion of flattened beams, the spatialized information relates to the off‐axis dose modulation due to both the incident fluence modulation, mainly caused by the flattening filter, and the difference in spectral energy response between water and a‐Si of the EPID.^37^ In addition, the Flood Field correction applied on the mPIs flattens the EPID signal. The intrinsic off‐axis modulation of the incident fluence is therefore completely erased from the input data.

**FIGURE 3:**
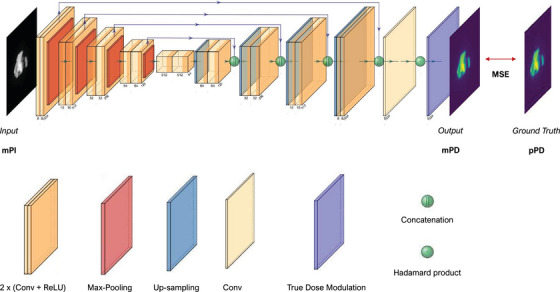
Model architecture containing the U‐net followed by the True Dose Modulation layer.

To address this in the present deep‐learning approach, different learning‐based solutions were explored such as densely connected, 2D locally connected and 2D CoordConv layer proposed by Liu et al.^36^ However, these layers must be optimized in a time‐consuming learning process, and they did not provide sufficient model convergence. A simpler and more modular solution was chosen. The method involves introducing a 2D non‐trainable layer in the model, called True Dose Modulation (TDM). This layer is computed outside the main learning phase from results of the primary trained CNN and specific reference data (see Section 2). The TDM is then added to the model as the last layer (see Figure 3). Thus, it contributes to the calculation of the Mean Squared Error (MSE) cost function to adjust CNN parameters through a fine‐tuning process.

### 2.3 Deep learning model

The proposed model is a U‐net followed by the TDM layer presented in the previous section. The overall model architecture is shown in Figure 3. In this work, hyperparameters such as number of kernels and convolutional layers as well as the model depth and loss function were manually tuned with a grid search procedure. The proposed U‐net has a depth of four max‐pooling layers with a stride of 2 × 2, where each block consists of two convolutional layers (Conv) with an identical number of kernels followed by a Rectifier Linear Unit (ReLU) activation function. After passing through the bottleneck, the signal is reconstructed by four up‐sampling layers with a stride of 2 × 2. The last decoder's block consists of three convolutional layers where the last one has a single kernel of shape 1 × 1 and no activation function. The purpose of this subsidiary layer is to perform a point‐wise convolution to recover the number of output channels, which is equal to one for the dose values. Skip‐connections were also added to facilitate signal reconstruction in the decoding‐path.^41^ These connections consist in concatenating the features maps of equal spatial dimensions coming out of encoder blocks to those going into decoder blocks. The kernel number of convolutional layers begins at eight and is doubled in each of the new blocks constituting the encoding‐path. In the same manner, this number is successively divided by two in the decoding‐path. Finally, the TDM layer is connected to the U‐net output through an Hadamard product to characterize the contribution of each pixel. This array has a resolution of 1024 × 1024 pixel and a single channel to keep the dimensions of output data. In this study, no dropout, batch normalization layer and cost function regularization term were used.

### 2.4 Training

**FIGURE 4:**
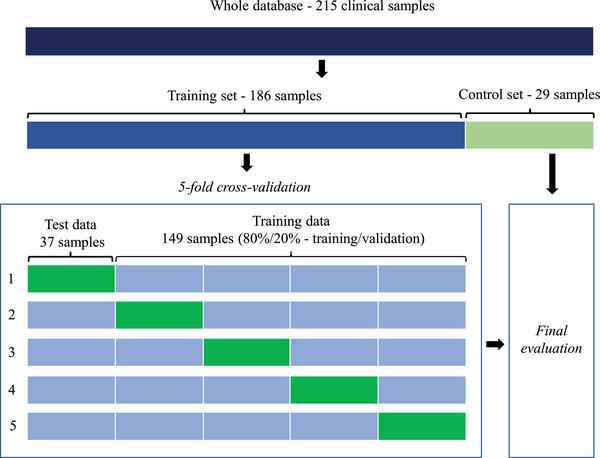
Data splitting steps.

The model was developed via a cross‐validation procedure. Five U‐nets with identical hyperparameters were randomly initialized and successively optimized on different combinations of training, validation, and test sets (see Figure 4). A proportion of 80% training set (119 samples) and 20% validation set (30 samples) was chosen. Each cross‐validation learning process was conducted in two stages. First, primary U‐nets were trained without TDM layer. Then, each TDM was computed as the ratio of the pPD of the 260 mm square beam to the corresponding inferred mPDs and added to the U‐net output as described in Figure 3. Finally, a fine‐tuning of all model parameters was performed on the same dataset. Once the five models were trained, the one that provided the lowest average ϒ‐index (2%‐2 mm > 10% D_max_) on its test dataset (37 samples) was selected for the final evaluation with the 29 clinical and six square control beams.

The Adam algorithm was used as the optimizer to minimize the MSE. Its parameters were set to 0.9, 0.999 and 10^−6^ for β1, β2 and ε, respectively. Adam optimizer is based on the stochastic gradient descent algorithm; it combines an adaptive learning rate and a second momentum.^42^ For the first and second training phases, the maximum learning rate was initially set to 10^−3^ and 10^−4^, respectively. If the validation loss did not decrease during four epochs, the learning rate was reduced by a factor of 0.8. Furthermore, to avoid overfitting and interrupt the training at the most appropriate time, an early stopping callback based on the validation loss and a delta set to five epochs was used. Each trained model was then saved with its best state, that is, the one providing the best performance in terms of loss on the validation data. In addition, a custom callback was also implemented to compute the average ϒ‐index and the average ϒ‐passing rate at each five epochs step. Conditioned by technical limitations, an amount of four samples was chosen for the training and validation batches. Weights and biases were initialized with Glorot uniform^43^ and zeros, respectively.

All processes including preprocessing, model architecture, trainings, and quantitative analysis were implemented in Python (version 3.8.10) with Tensorflow (version 2.0.1) and Keras (version 2.1.0) as backend. Each training was performed using a single job of the Vertex‐AI API from the Google Cloud Computing with an NVIDIA Tesla P4 GPU and 16 GB of CPU memory. The inferences were performed using no GPU and an Intel Xeon CPU with 12 cores clocked to 3.5 GHz.

### 2.5 Model evaluation

#### 2.5.1 Amount of training data

A study was conducted to assess the performance of the proposed model based on the amount of training data provided. For this purpose, the training method described in the previous section was repeated ten times while keeping the model architecture (see Sections 3 and 4) but increasing the amount of training data from 18 to 186 samples (18, 37, 56, 74, 93, 112, 130, 149, 168 and 186 samples). Note that, hyperparameters were preliminarily optimized for the maximum amount of data available, that is, 186 training samples. At each fold of the cross‐validation of each dataset, the samples were randomly selected and the proportion of 80%/20% between training and validation data was kept. Then, a U‐net was trained to convert mPIs into mPDs with the two‐step training method. The γ‐index statistics (2%‐2 mm > 10% D_max_) were computed between the mPDs and pPDs with the 29 clinical control beams (see Section 2.1). The statistics obtained for each dataset aggregate the results from the five U‐nets trained on that same set.

#### 2.5.2 Training

For the 186 training samples set, the cross‐validation boxplots of the averages γ‐index (2%‐2 mm > 10% D_max_) and γ‐passing rate were plotted to determine which model performed best. Each box plot aggregates results of a single cross‐validation model with its test set.

The convergence of the retained model over epochs was assessed by analyzing its learning phase. The records contained the training and validation losses, the learning rate, and the averages of the γ‐index and γ‐passing rate computed between mPDs and pPDs of all clinical control beams.

#### 2.5.3 TDM and square beams

To qualitatively assess the contribution of the TDM layer and the performance of the selected model on elementary cases, an analysis of dose profiles was performed on three square beams of 15 mm, 100 mm, and 200 mm side. For each beam, the left‐right profiles centered to the beam axis of the absolute mPDs computed by the models with (U‐net_TDM_) and without TDM layer (U‐net) were compared to those of the corresponding reference pPDs. For didactic purpose, the left‐right profiles of grayscale mPIs and that of the TDM layer were also plotted.

#### 2.5.4 Model performance

The overall performance of the model was assessed through a quantitative analysis based on the six square beams and the 29 IMRT S&S control beams (see Section 2.1). The statistics of ϒ‐index (2%‐2 mm > 10% D_max_), ϒ‐passing rate, and local absolute and global relative errors were computed between the pPDs and the mPDs of the U‐net, U‐net_TDM_ and the analytical conversion algorithm (Analytic). To facilitate this analysis, box plot of each model and metric with the clinical control beams was plotted.

Using the IMRT S&S control beams set, a visual analysis of the results obtained with six different tumor locations was performed. For this purpose, the pPDs and mPDs inferred by the U‐net_TDM_ as well as their respective left‐right profiles centered on the beam axis were plotted. The ϒ‐index map, the averages of ϒ‐index and ϒ‐passing rate were also computed.

## 3 RESULTS

### 3.1 Amount of training data

**FIGURE 5:**
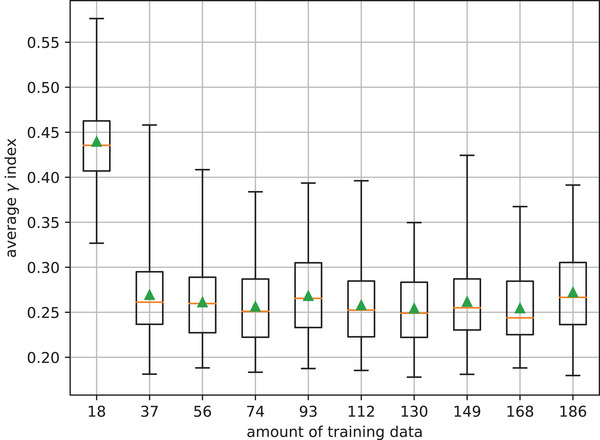
Average γ‐index box plots computed between pPDs and mPDs inferred by the five U‐nets trained in each training set.

The Figure 5 represents the box plots of the average γ‐index as a function of the amount of training data. For the dataset containing 18 samples, an average γ‐index of 0.43 (±0.06) and a maximum average γ‐index of 0.58 were obtained. These results were significatively improved from the 37 training samples set where an average γ‐index of 0.27 (±0.05) was obtained. From this set, the results were roughly equivalent in terms of mean, median and interquartile range. For datasets containing between 37 and 186 training samples, the means γ‐indexes were between 0.27 (±0.05) and 0.25 (±0.05) for an average of 0.26 (±0.05). In this interval, the maximum averages γ‐indexes did not exceed 0.46. Finally, the best results were obtained by the 168 training samples set, which provided a median of 0.24 and an average γ‐index of 0.25 (±0.05).

### 3.2 Training

**FIGURE 6:**
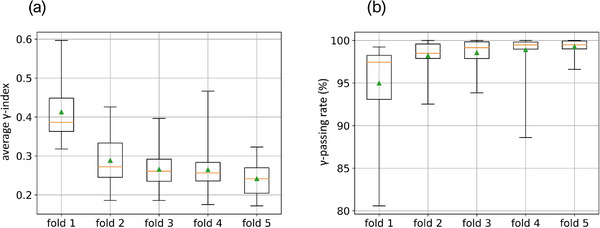
Average ϒ‐index (a) and average ϒ‐passing rate (b) box plots computed on each test set of each cross‐validation model.

The results of the five cross‐validation U‐nets trained from the set of 186 samples are shown in Figure 6. As a reminder, the learnings are decorrelated from each other. The training and test data sets per fold were randomly constructed while keeping the same number of samples to cover the entire database. For all cases, the average γ‐index was less than 0.29 (±0.08). The minimum average γ‐index was 0.24 (±0.04) and the maximum average γ‐passing rate was 99.28 (±0.84)%. These results were obtained by the model associated with the 5^th^ fold; it was thus retained for the continuation of the study. Note that the interquartile ranges of the γ‐passing rate were between 0.47% (fold 5) and 4.35% (fold 1). These dispersions highlight the importance of using the cross‐validation procedure to determine which model performs best.

**FIGURE 7:**
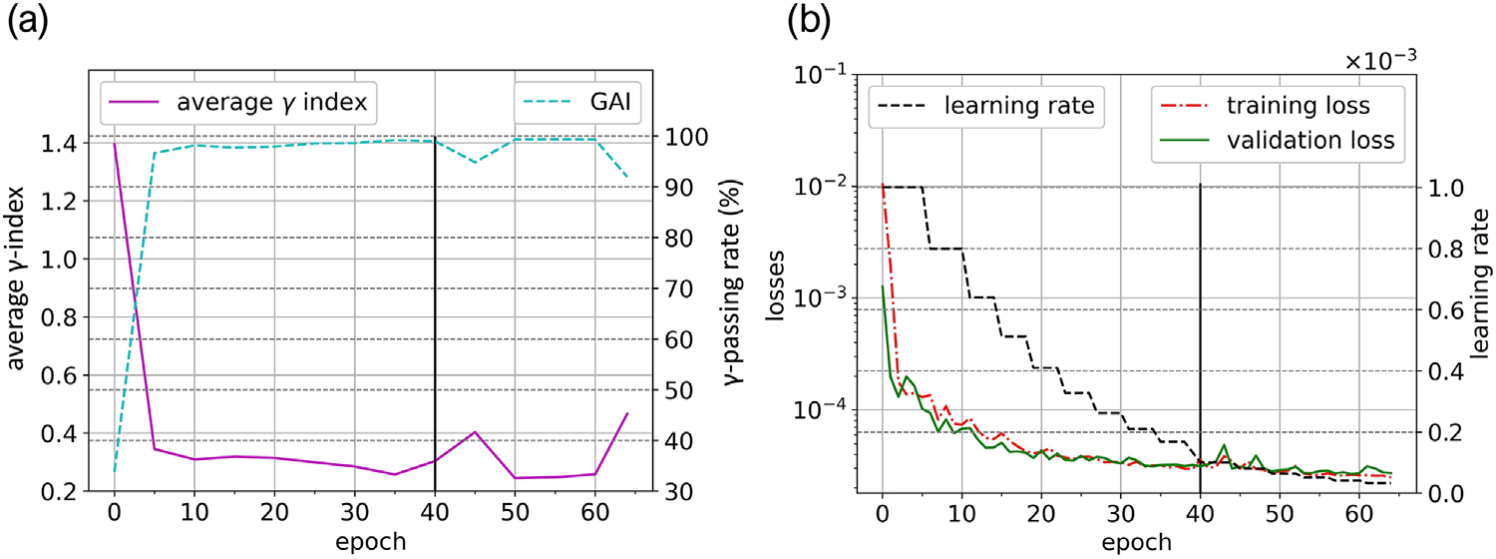
Training history of the 5^th^ model composed of the learning rate, the training and validation losses (a), the average γ‐index and the average ϒ‐passing rate (b). On the two graphs, at the 40^th^ epoch (vertical line), the TDM was computed and added to the U‐net output.

The learning rate, training and validation losses, γ‐index, and γ‐passing rate over epochs of the selected model are shown in Figure 7. Based on the validation loss, the first learning phase was interrupted at the 40^th^ epoch. At this time, the model has provided results of 0.30 (±0.17) and 98.99 (±0.82)% for the averages of the γ‐index and the γ‐passing rate, respectively. Due to the addition of the TDM layer to the U‐net output, a slight increase in losses was observed. This performance degradation was subsequently reduced by the fine‐tuning process.

Since the lowest validation loss was obtained by the 59^th^ epoch model state, it was selected for the remainder of this study. This choice was also reinforced by the degradation of all metrics starting at the 60^th^ epoch. This model state provided averages of 0.28 (±0.19) and 99.09 (±0.75)% for the γ‐index and the γ‐passing rate, respectively. Given the technical specifications described in Section 1.D, the entire training lasted approximately 35 min and the fine‐tuning was completed in less than 15 min.

### 3.3 TDM and square beams

**FIGURE 8:**
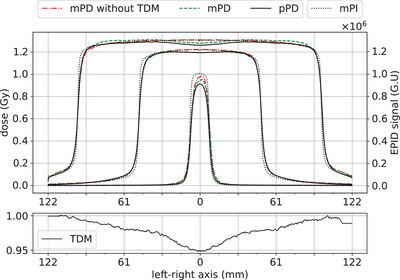
Left‐right profiles centered on the beam axis of mPIs in grayscale units (G.U), TDM (bottom graph), pPDs, and mPDs of the U‐net and U‐net_TDM_ for square beams of 15 mm, 100 mm, and 200 mm side.

The Figure 8 displays the left‐right profiles of mPIs, pPDs, mPDs of U‐net, U‐net_TDM_, and the TDM. Regarding the penumbra width of mPIs, both U‐nets have well recovered that of the reference pPDs. This is particularly visible at the bottom and top of all penumbras. With respect to the off‐axis dose modulation, better agreement was obtained by the model coupled with a TDM layer compared to the model without it. This is corroborated by the horn effect that is clearly visible on TDM, mPD and pPD of the 200 mm square beam. However, discrepancies of approximately 3% are observed on the top of profiles for the 15 mm and 200 mm square beams. These deviations are overall larger with the U‐net alone, except at the edges of the largest beam. Note that the linearity observed on the first and last millimeters of the TDM profile is due to the data preprocessing (see Section 2.1).

### 3.4 Model performance

**FIGURE 9:**
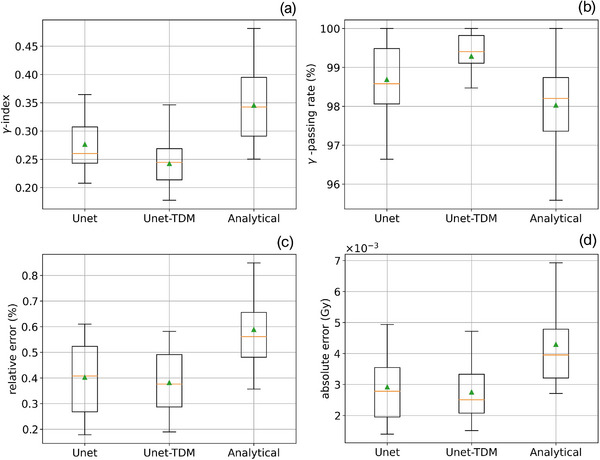
ϒ‐index (a), ϒ‐passing rate (b), absolute (c) and relative (d) errors box plots computed between pPDs and mPDs of U‐net, U‐net_TDM_ and analytical conversion models with the 29 IMRT S&S control set.

The Table 1 gathers the statistics obtained by the U‐net, U‐net_TDM_ and analytical conversion model (Analytic) on the whole control database. Globally, for the 29 IMRT S&S beams, a very good agreement between pPDs and mPDs of all models is observed. Indeed, with the restrictive criterion used (2%‐2 mm > 10% D_max_), a maximum average γ‐index less than 0.34 (±0.06) and a minimum average γ‐passing rate greater than 98.02 (±1.23)% were obtained. Maximum averages of 0.43 × 10^−2^ (±0.13 × 10^−2^) Gy and 0.58 (±0.13)% were obtained for the absolute and relative dose errors, respectively. These results were obtained by the analytical model and those of the two U‐nets were systematically better. For instance, differences of 0.10 (*p* < 0.001) and 1.27% (*p* < 0.001) are observed in favor to the U‐net_TDM_ for the average γ‐index and average γ‐passing rate, respectively. For the average absolute and relative dose errors, the differences are approximately 0.16 × 10^−2^ Gy (*p* < 0.001) and 0.2% (*p* < 0.001), respectively. This trend is also visible on the box plots of the models obtained with the clinical beams in Figure 9. For all metrics, the averages and medians are in favor to the U‐net_TDM_ and the interquartile ranges are lower except for the relative error. About the square beams, the performance of the U‐net_TDM_ remains superior to that of the analytical model. Overall, a decrease in the performance of the three models is observed with the square beams compared to the clinical beams.

**TABLE 1:** Statistical results between pPDs and mPDs of U‐net, U‐net_TDM_ and analytical model for each control dataset type

		5 Square beams			29 IMRT S&S beams		
Metric	Model	min	mean (±SD)	max	min	mean (±SD)	max
γ‐index	U‐net^a^	0.22	0.36 (±0.11)	0.58	0.21	0.28 (±0.04)	0.36
	U‐net_TDM_ ^b^	0.19	0.31 (±0.16)	0.63	0.18	0.24 (±0.04)	0.35
	Analytic^c^	0.25	0.37 (±0.11)	0.53	0.25	0.34 (±0.06)	0.48
γ‐passing rate (%)	U‐net^a^	74.77	92.69 (±8.21)	97.95	96.64	98.69 (±0.96)	100.00
	U‐net_TDM_ ^b^	93.80	98.83 (±2.40)	100.00	97.40	99.29 (±0.70)	100.00
	Analytic^c^	79.59	96.24 (±8.20)	100.00	95.58	98.02 (±1.23)	100.00
Absolute error × 10^−2^ (Gy)	U‐net^a^	0.10	0.76 (±0.56)	1.68	0.14	0.29 (±0.11)	0.61
	U‐net_TDM_ ^b^	0.09	0.65 (±0.67)	1.90	0.15	0.27 (±0.10)	0.56
	Analytic^c^	0.21	0.63 (±0.31)	1.02	0.27	0.43 (±0.13)	0.80
Relative error (%)	U‐net^a^	0.11	0.50 (±0.33)	1.07	0.18	0.40 (±0.13)	0.61
	U‐net_TDM_ ^b^	0.01	0.52 (±0.51)	1.47	0.18	0.38 (±0.11)	0.58
	Analytic^c^	0.21	0.53 (±0.21)	0.79	0.36	0.58 (±0.13)	0.98

^a^ U‐net without TDM layer

^b^ U‐net coupled with its TDM layer and fine‐tuned

^c^ Analytical mPI‐to‐mPD conversion model from EPIbeam system.

Regarding the contribution of the TDM layer, we note that adding it to the U‐net structure provides better results. Indeed, for all metrics and data types, results of the Unet_TDM_ were better compared to the U‐net alone. With clinical control beams, differences of 0.04 (*p* < 0.001) and 0.6% (*p* < 0.001) were obtained in favor of the Unet_TDM_ for the average γ‐index and average γ‐passing rate, respectively. This is corroborated on all box plots in Figure 9 where medians and interquartile ranges are in favor to the Unet_TDM_. The benefit of the TDM layer was also reinforced with the six square beams with an increase of approximately 6% (*p*‐value non‐significant) of the average γ‐passing rate and a decreasing of 0.05 of the γ‐index (*p*‐value non‐significant) by the Unet_TDM_. These discrepancies between both U‐nets are even more significant on the minimum averages of the different metrics. For instance, a difference of approximately 19 % was obtained between the minimum averages of the γ‐passing rate of both U‐nets.

**FIGURE 10:**
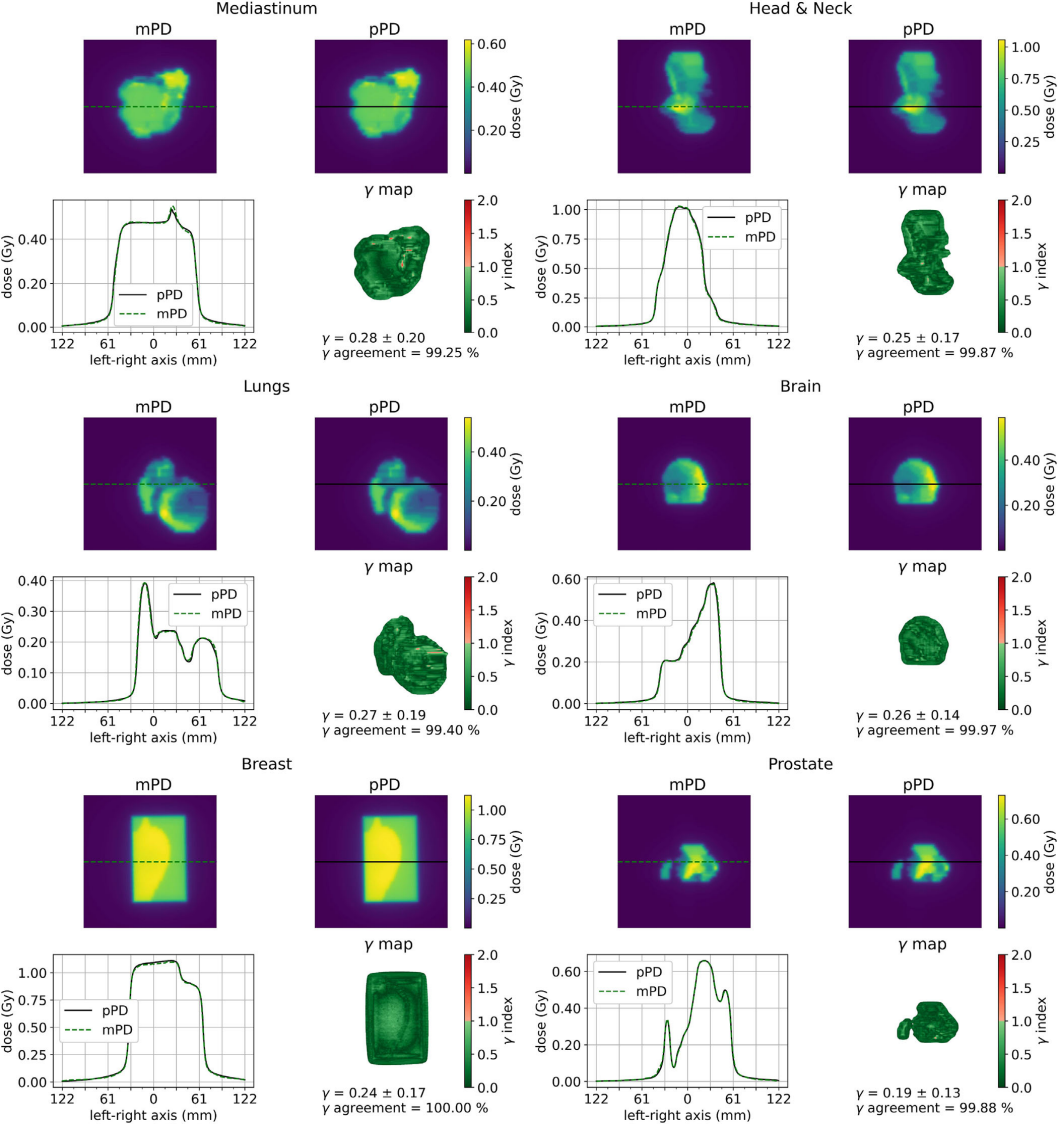
pPDs, mPDs of the U‐net_TDM_, ϒ map and left‐right dose profiles centered on the beam axis of six clinical treatment beams from the 29 IMRT S&S control set.

The Figure 10 illustrates an overview of results obtained by the U‐net_TDM_ with six beams from the clinical control dataset. On all profiles, very good agreement between mPDs and pPDs was obtained. This is corroborated by the analysis of the γ‐index maps, where most pixels obtained a value below 0.75. For all cases, the average γ‐index was less than 0.28 (±0.20) and the γ‐passing rate was greater than 99.25%.

## 4 DISCUSSION

In previous studies, MLPs were trained to convert mPI patches into mPD pixels from a set of predefined input features.^23^, ^24^, ^25^ In this study, a U‐net was developed to convert, in a single forward pass, entire grayscale mPIs into absolute mPDs of IMRT S&S beams of various tumor sites.

The benefit of the TDM layer was directly visible on the analysis of the dose profiles illustrated in Figure 8. Indeed, for all cases, the horn effect was well recovered by the U‐net_TDM_ compared to the U‐net alone. The loss record illustrated in Figure 7 demonstrated the need to fine‐tune the model after the TDM addition to increase its accuracy. The benefits of the TDM layer on the overall performance was also reinforced by the quantitative analysis. For the 29 IMRT S&S control beams, a slight increase in the performance of the U‐net_TDM_ was observed. Regarding the square beams, this trend was even more significant where an increase of approximately 6% of the average γ‐passing rate was obtained. Overall, we note that the use of the TDM layer in combination with the fine‐tuning improves the results on all tested data types. An interesting point to note is that the TDM profile in Figure 8 has similar shape and amplitude to those expected for flattened beams. Thus, this layer appears to be equivalent to the Off‐Axis‐Ratio correction usually encountered in analytical models.^44^ As expected, the TDM recovers the spatialized information that could not be encoded by the pretrained U‐net.

In clinical routine, tolerance limits of the γ‐passing rate are typically set at 95% with the criteria of 3%‐2 mm > 10% D_max_^1^. In this study, the restrictive criteria of 2%‐2 mm > 10% D_max_ were used, and the γ‐passing rates obtained by the U‐net_TDM_ with the 29 clinical control beams were systematically greater than 97.4%. Specifically, with respect to the penumbra width, very good agreement between the mPDs of U‐nets and pPDs in high and low dose regions was observed. It should be noted that slightly worse results were obtained with the square beams comparatively to the IMRT S&S beams set. This can be explained by the fact that U‐nets were exclusively trained on clinical beams. In addition, the distance to agreement criterion of the γ‐index tends to improve the results for more modulated dose distributions.^45^

In this study, several assumptions were made. First, the pPDs computed by the prediction algorithm were used as reference model outputs. This solution was preferred over the TPS DICOM RT dose files because it facilitates the data collection while having a sufficient accuracy. Second, we assumed that training the U‐nets with the computed pPDs rather than the analytical mPDs avoids any additional bias related to the calculations of the analytical conversion algorithm. This choice is reinforced by the fact that the latter is, like the U‐net, parametrized from a set of mPIs and pPDs computed by the prediction algorithm. Furthermore, although the mPIs necessarily incorporate the fluctuations inherent in normal equipment operation, an effort was made to rigorously ensure the absence of delivery errors in the treatment plans used. In this way, we hope to eliminate any bias in the dataset and ensure achievable accuracy by the U‐net enabling to detect clinically relevant errors. It is important to note that since the U‐net was trained on TPS‐based reference data, it is not intended for TPS modeling validation. Any systematic errors in TPS modeling will necessarily be incorporated into the model learning and, consequently, into the model itself. In this way, the part of the patient QA process is limited to detecting LINAC delivery errors relative to the expected beams. Finally, in contrast to previous studies,^23^, ^24^, ^25^ it was assumed that the tumor sites DICOM tag should not be made as a specific learning feature. Therefore, no distinction was made in the construction of the datasets.

The final model was trained with a set of 186 samples. This amount of training data may seem small compared to those typically found in DL applications. However, the results of the study described in Section 3. have shown that sufficient model accuracy can be achieved with approximately five times fewer samples than those used for the final model. As presented in Figure 5, U‐nets trained with the dataset of 37 samples provided a maximum average γ‐index of 0.46 on the clinical control beams, and the results for datasets with a larger number of samples were roughly equivalent. Although these results are satisfying, they were obtained with only five models per dataset. Therefore, it could be interesting to increase the number of trained models. This study could also be extended to larger datasets to determine if higher accuracy can be achieved.

Overall, better agreement was obtained with the mPDs of U‐nets than those of the analytical conversion model. This is corroborated by the quantitative analysis and the Figure 9 where the U‐net_TMD_ provides the best results for all metrics and all tested data types. This gain in accuracy of the U‐net_TMD_ over the analytical model show that it could be more sensitive in detecting delivery errors. A long‐term clinical study could be interesting to quantify this sensitivity. Computation time measurements on suitable hardware could also determine whether DL methods are an interesting alternative to existing methods for EPID‐based non‐transit dosimetry.

However, although the results show a gain in accuracy of U‐net, the objective of this study is not to replace the existing methods but to extend previous studies using MLPs. In this work, we investigated the feasibility of using the popular U‐net for the mPI‐to‐mPD conversion and provided a method to better recover the spatialized information. More prospectively, this work was a first step towards the development of a DL model for EPID‐based dosimetry in transit conditions. Indeed, the use of EPID for in vivo dosimetry is of major interest for patient‐specific QA.^46^ As radiotherapy requires increasingly precise and rapid QA control systems, the achievable accuracy and possible computational speed of DL models^47^ make them potential candidates for the development of an efficient real‐time tool for in vivo dosimetry.

As a reminder, the present study was limited to IMRT S&S beams obtained from one linac model, one a‐Si‐EPID model and one energy. Since the EPID is energy dependent^48^ and there is an inherent variability in response between imagers, it appears that the operating range of the proposed U‐net is limited by the energy, fluence mode, and linac and EPID models used for its training. Regarding treatment technics, it can be assumed that the model should provide acceptable performance since the steps to convert mPIs to mPDs remain the same. In this sense, it might be interesting to analyze the results with other treatment technics and to extend the study to other beams (energy, fluence mode and equipment).

In this work, the feasibility of using DL models for EPID‐based dosimetry in non‐transit conditions was shown. The results show an accuracy of the proposed model at least equivalent to existing methods.

## 5 CONCLUSIONS

A deep learning‐based model was developed to convert portal images into absolute dose distributions of IMRT S&S beams. The method consists of optimizing a U‐net followed by the True Dose Modulation layer using a two‐step learning process. With this architecture, the model can learn the global features and recover the intrinsic dose modulation of flattens beams. The accuracy obtained shows that this method has great potential for EPID‐based non‐transit dosimetry.

## AUTHOR CONTRIBUTIONS

Xavier Franceries, François Husson and Marie‐Véronique Le Lann devised, planned, and directed the study. François Husson also performed the EPID acquisitions. Lucas Dal Bosco designed the training method and models, performed the experiment, and drafted the manuscript with support from Blandine Romain, François Smekens and François Husson. All authors discussed the results and contributed to the final manuscript.

## CONFLICT OF INTEREST

L. Dal Bosco, F. Husson, B. Romain, and F. Smekens are employees of Dosisoft SA. The other authors do not have any relevant conflicts of interest to disclose.

## Data Availability

The data that support the findings of this study are available on request from the corresponding author. The data are not publicly available due to privacy and ethical restrictions.

## APPENDIX A

### A.1

**TABLE 2:** Description of plans used for the training dataset

Plan number	Tumor site	Dose per fraction (Gy)	Number of beams	Average number of segments per beam	Dose point prescription (MUs/Gy)	Plan MCS	Average γ‐passing rate^c^ (%)	Number of beams used
1	H&N**^a^**	0.140	7	9	289.20	0.67	98.63	5
2	H&N**^a^**	0.142	7	13	420.49	0.47	97.59	5
3	H&N**^a^**	0.058	7	5	256.77	0.57	99.35	7
4	Prostate	0.054	7	6	252.43	0.70	98.99	6
5	Prostate	0.109	7	14	347.67	0.49	99.19	6
6	Prostate	0.158	7	7	198.68	0.66	98.88	6
7	Prostate	0.109	7	14	374.38	0.48	97.99	7
8	H&N^a^	0.062	6	8	254.37	0.56	98.47	5
9	Stomach	0.074	7	8	328.19	0.49	98.75	6
10	Lungs	0.070	5	10	450.10	0.54	99.46	4
11	Breast	0.080	2	3	98.69	N.A.^b^	99.68	2
12	H&N**^a^**	0.083	5	7	180.97	0.73	99.27	5
13	Lungs	0.062	6	9	275.43	0.65	98.45	6
14	H&N**^a^**	0.083	6	9	1267.11	0.63	98.94	6
15	Kidney	0.318	7	5	177.75	0.73	99.49	7
16	Brain	0.055	6	5	208.83	0.63	98.09	5
17	Prostate	0.189	6	8	325.79	0.73	99.42	6
18	H&N^a^	0.119	7	14	515.25	0.44	98.82	7
19	Prostate	0.060	7	7	262.91	0.66	99.31	5
20	H&N	0.063	5	7	185.18	0.63	99.27	4
21	H&N^a^	0.137	5	7	207.39	0.70	99.00	4
22	Mediastinum	0.061	7	7	890.04	0.64	98.79	6
23	H&N^a^	0.139	5	9	230.67	0.71	98.65	4
24	Lungs	0.116	7	4	171.74	0.70	99.01	3
25	H&N^a^	0.207	5	11	217.35	0.68	97.80	3
26	Prostate	0.090	7	8	259.73	0.55	98.79	7
27	H&N^a^	0.139	7	6	171.51	0.71	98.46	7
28	Breast	0.079	2	4	107.77	0.00	99.55	1
29	Brain	0.188	7	8	197.93	0.67	98.68	6
30	Prostate	0.056	7	6	309.44	0.69	98.81	6
31	H&N^a^	0.077	5	7	505.42	0.68	98.45	4
32	Rectum	1.055	5	19	247.43	0.60	99.14	4
33	H&N^a^	0.144	7	13	386.35	0.45	98.10	6
34	Prostate	0.155	7	8	195.62	0.73	99.25	5
35	Stomach	0.073	7	8	338.63	0.51	99.22	5
36	Rectum	0.082	7	8	276.41	0.55	99.50	5

^a^ Head and Neck tumor site

^b^ Non‐Applicable values of MCS score^49^ are caused by a specific case where all MLC leaves of a bank are aligned

^c^ The average γ‐passing rate is computed on all beams of the plan with the criteria of 2%‐2 mm >10% D_max_.

**TABLE 3:** Description of plans used for the control dataset

Plan number	Tumor site	Dose per fraction (Gy)	Number of beams	Average number of segments per beam	Dose point prescription (MUs/Gy)	MCS score	Average γ‐passing rate^c^ (%)	Number of beams used
1	Breast	0.082	2	3	93.25	N.A.^b^	99.37	2
2	Prostate	0.187	6	8	378.05	0.63	98.02	2
3	Prostate	0.106	7	10	266.64	0.51	98.97	1
4	Mediastinum	0.306	7	10	208.15	0.30	98.11	7
5	Brain	0.187	6	6	206.34	0.69	97.71	6
6	H&N^a^	0.213	5	12	230.48	0.60	97.57	5
7	Lungs	0.068	6	8	270.57	0.58	98.17	6

^a^ Head and Neck tumor site.

^b^ Non‐Applicable values of MCS score^49^ are caused by a specific case where all MLC leaves of a bank are aligned.

^c^ The average γ‐passing rate is computed on all beams of the plan with the criteria of 2%‐2mm > 10% D_max_.

## References

[1] Miften M , Olch A , Mihailidis D , et al. Tolerance limits and methodologies for IMRT measurement‐based verification QA: recommendations of AAPM Task Group No. 218. Med Phys. 2018;45(4):e53‐e83. doi:10.1002/mp.12810 29443390

[2] Nijsten SMJJG , Minken AWH , Lambin P , Bruinvis IAD . Verification of treatment parameter transfer by means of electronic portal dosimetry. Med Phys. 2004;31(2):341‐347. doi:10.1118/1.1640972 15000620

[3] Louwe RJW , McDermott LN , Sonke JJ , et al. The long‐term stability of amorphous silicon flat panel imaging devices for dosimetry purposes. Med Phys. 2004;31(11):2989‐2995. doi:10.1118/1.1803751 15587651

[4] McDermott LN , Louwe RJW , Sonke JJ , van Herk MB , Mijnheer BJ . Dose‐response and ghosting effects of an amorphous silicon electronic portal imaging device. Med Phys. 2004;31(2):285‐295. doi:10.1118/1.1637969 15000614

[5] Bailey DW , Kumaraswamy L , Bakhtiari M , Malhotra HK , Podgorsak MB . EPID dosimetry for pretreatment quality assurance with two commercial systems. J Appl Clin Med Phys. 2012;13(4):3736. doi:10.1120/jacmp.v13i4.3736 22766944 PMC5716510

[6] Steciw S , Warkentin B , Rathee S , Fallone BG . Three‐dimensional IMRT verification with a flat‐panel EPID. Med Phys. 2005;32(2):600‐612. doi:10.1118/1.1843471 15789607

[7] Renner WD , Norton K , Holmes T . A method for deconvolution of integrated electronic portal images to obtain incident fluence for dose reconstruction. J Appl Clin Med Phys. 2005;6(4):22‐39. doi:10.1120/jacmp.v6i4.2104 PMC5723452 16421498

[8] Low DA , Harms WB , Mutic S , Purdy JA . A technique for the quantitative evaluation of dose distributions. Med Phys. 1998;25(5):656‐661. doi:10.1118/1.598248 9608475

[9] Van Esch A , Depuydt T , Huyskens DP . The use of an aSi‐based EPID for routine absolute dosimetric pre‐treatment verification of dynamic IMRT fields. Radiother Oncol J Eur Soc Ther Radiol Oncol. 2004;71(2):223‐234. doi:10.1016/j.radonc.2004.02.018 15110457

[10] Lee C , Menk F , Cadman P , Greer PB . A simple approach to using an amorphous silicon EPID to verify IMRT planar dose maps. Med Phys. 2009;36(3):984‐992. doi:10.1118/1.3075817 19378759

[11] Nicolini G , Fogliata A , Vanetti E , Clivio A , Cozzi L . GLAaS: An absolute dose calibration algorithm for an amorphous silicon portal imager. Applications to IMRT verifications: GLAaS IMRT verification. Med Phys. 2006;33(8):2839‐2851. doi:10.1118/1.2218314 16964860

[12] Warkentin B , Steciw S , Rathee S , Fallone BG . Dosimetric IMRT verification with a flat‐panel EPID. Med Phys. 2003;30(12):3143‐3155. doi:10.1118/1.1625440 14713081

[13] Pasma KL , Dirkx ML , Kroonwijk M , Visser AG , Heijmen BJ . Dosimetric verification of intensity modulated beams produced with dynamic multileaf collimation using an electronic portal imaging device. Med Phys. 1999;26(11):2373‐2378. doi:10.1118/1.598752 10587219

[14] Miri N , Keller P , Zwan BJ , Greer P . EPID‐based dosimetry to verify IMRT planar dose distribution for the aS1200 EPID and FFF beams. J Appl Clin Med Phys. 2016;17(6):292‐304. doi:10.1120/jacmp.v17i6.6336 27929502 PMC5690494

[15] Han B , Ding A , Lu M , Xing L . Pixel response‐based EPID dosimetry for patient specific QA. J Appl Clin Med Phys. 2017;18(1):9‐17. doi:10.1002/acm2.12007 PMC5393354 28291939

[16] Seneclauze A , Boissard P , Baudier T , Dupuis P , Malet C . 9 MLC error detection with EPIBeam for VMAT patient quality assurance. Phys Medica Eur J Med Phys. 2018;56:6. doi:10.1016/j.ejmp.2018.09.022

[17] El Naqa I , Ruan D , Valdes G , et al. Machine learning and modeling: data, validation, communication challenges. Med Phys. 2018;45(10):e834‐e840. doi:10.1002/mp.12811 30144098 PMC6181755

[18] Valdes G , Scheuermann R , Hung CY , Olszanski A , Bellerive M , Solberg TD . A mathematical framework for virtual IMRT QA using machine learning. Med Phys. 2016;43(7):4323. doi:10.1118/1.4953835 27370147

[19] Carlson JNK , Park JM , Park SY , Park JI , Choi Y , Ye SJ . A machine learning approach to the accurate prediction of multi‐leaf collimator positional errors. Phys Med Biol. 2016;61(6):2514‐2531. doi:10.1088/0031-9155/61/6/2514 26948678

[20] Guidi G , Maffei N , Vecchi C , et al. A support vector machine tool for adaptive tomotherapy treatments: Prediction of head and neck patients criticalities. Phys Medica PM Int J Devoted Appl Phys Med Biol Off J Ital Assoc Biomed Phys AIFB. 2015;31(5):442‐451. doi:10.1016/j.ejmp.2015.04.009 25958225

[21] Valdes G , Chan MF , Lim SB , Scheuermann R , Deasy JO , Solberg TD . IMRT QA using machine learning: a multi‐institutional validation. J Appl Clin Med Phys. 2017;18(5):279‐284. doi:10.1002/acm2.12161 28815994 PMC5874948

[22] Jia M , Wu Y , Yang Y , et al. Deep learning‐enabled EPID‐based 3D dosimetry for dose verification of step‐and‐shoot radiotherapy. Med Phys. 2021;48(11):6810‐6819. doi:10.1002/mp.15218 34519365

[23] Kalantzis G , Vasquez‐Quino LA , Zalman T , Pratx G , Lei Y . Toward IMRT 2D dose modeling using artificial neural networks: a feasibility study. Med Phys. 2011;38(10):5807‐5817. doi:10.1118/1.3639998 21992395

[24] Mahdavi SR , Bakhshandeh M , Rostami A , Arfaee AJ . 2D Dose Reconstruction by Artificial Neural Network for Pretreatment Verification of IMRT Fields. J Med Imaging Radiat Sci. 2018;49(3):286‐292. doi:10.1016/j.jmir.2018.05.004 32074055

[25] Chatrie F , Franceries X , Le Lann MV . Deep learning for regression problem applied to radiotherapy pre‐treatment verification .; 2019:1‐6.

[26] Lecun Y , Bottou L , Bengio Y , Haffner P . Gradient‐based learning applied to document recognition. Proc IEEE. 1998;86:2278‐2324. doi:10.1109/5.726791

[27] Ronneberger O , Fischer P , Brox T . U‐net: convolutional networks for biomedical image segmentation. *arXiv*. 2015. doi:10.48550/arXiv.1505.04597

[28] Barragan‐Montero AM , Nguyen D , Lu W , et al. Three‐dimensional dose prediction for lung IMRT patients with deep neural networks: robust learning from heterogeneous beam configurations. Med Phys. 2019;46(8):3679‐3691. doi:10.1002/mp.13597 31102554

[29] Lee MS , Hwang D , Kim JH , Lee JS . Deep‐dose: a voxel dose estimation method using deep convolutional neural network for personalized internal dosimetry. Sci Rep. 2019;9(1):10308. doi:10.1038/s41598-019-46620-y 31311963 PMC6635490

[30] Xing Y , Zhang Y , Nguyen D , Lin MH , Lu W , Jiang S . Boosting radiotherapy dose calculation accuracy with deep learning. J Appl Clin Med Phys. 2020;21(8):149‐159. doi:10.1002/acm2.12937 32559018 PMC7484829

[31] Keal J , Santos A , Penfold S , Douglass M . Radiation Dose Calculation in 3D Heterogeneous Media Using Artificial Neural Networks. Med Phys. 2021;48(5):2637‐2645. doi:10.1002/mp.14780 33595104

[32] Tsekas G , Bol GH , Raaymakers BW , Kontaxis C . DeepDose: a robust deep learning‐based dose engine for abdominal tumours in a 1.5 T MRI radiotherapy system. Phys Med Biol. 2021;66(6):065017. doi:10.1088/1361-6560/abe3d1 33545708

[33] Liu Y , Zhang Y . Low‐dose CT restoration via stacked sparse denoising autoencoders. Neurocomputing. 2018;284:80‐89. doi:10.1016/j.neucom.2018.01.015

[34] Olaciregui‐Ruiz I , Torres‐Xirau I , Teuwen J , van der Heide UA , Mans A . A Deep Learning‐based correction to EPID dosimetry for attenuation and scatter in the Unity MR‐Linac system. Phys Medica PM Int J Devoted Appl Phys Med Biol Off J Ital Assoc Biomed Phys AIFB. 2020;71:124‐131. doi:10.1016/j.ejmp.2020.02.020 32135486

[35] Chen S , Quan H , Qin A , Yee S , Yan D . MR image‐based synthetic CT for IMRT prostate treatment planning and CBCT image‐guided localization. J Appl Clin Med Phys. 2016;17(3):236‐245. doi:10.1120/jacmp.v17i3.6065 27167281 PMC5690904

[36] Liu R , Lehman J , Molino P , et al. An Intriguing Failing of Convolutional Neural Networks and the CoordConv Solution. ArXiv180703247 Cs Stat. Published online December 3, 2018. Accessed July 10, 2020. http://arxiv.org/abs/1807.03247

[37] Rowshanfarzad P , McCurdy BMC , Sabet M , Lee C , O'Connor DJ , Greer PB . Measurement and modeling of the effect of support arm backscatter on dosimetry with a varian EPID. Med Phys. 2010;37(5):2269‐2278. doi:10.1118/1.3369445 20527561

[38] Kauderer‐Abrams E . Quantifying TRANSLATION‐INVARIANCE IN CONVOLUTIONAL NEURAL NETworks. Published online December 10, 2017. doi:10.48550/arXiv.1801.01450

[39] Kayhan OS , van Gemert JC . On Translation Invariance in CNNs: Convolutional Layers can Exploit Absolute Spatial Location. ArXiv200307064 Cs Eess. Published online May 30, 2020. Accessed February 25, 2021. http://arxiv.org/abs/2003.07064

[40] Islam MA , Jia S , Bruce NDB . How Much Position Information Do Convolutional Neural Networks Encode? Published online January 22, 2020. doi:10.48550/arXiv.2001.08248

[41] Drozdzal M , Vorontsov E , Chartrand G , Kadoury S , Pal C . The Importance of Skip Connections in Biomedical Image Segmentation. ArXiv160804117 Cs. Published online September 22, 2016. Accessed November 24, 2021. http://arxiv.org/abs/1608.04117

[42] Kingma DP , Ba J . Adam: A Method for Stochastic Optimization. ArXiv14126980 Cs. Published online January 29, 2017. Accessed July 10, 2020. http://arxiv.org/abs/1412.6980

[43] Glorot X , Bengio Y . Understanding the difficulty of training deep feedforward neural networks. In: Proceedings of the Thirteenth International Conference on Artificial Intelligence and Statistics . JMLR Workshop and Conference Proceedings; 2010:249‐256. Accessed April 13, 2021. http://proceedings.mlr.press/v9/glorot10a.html

[44] Greer PB , Cadman P , Lee C , Bzdusek K . An energy fluence‐convolution model for amorphous silicon EPID dose prediction. Med Phys. 2009;36(2):547‐555. doi:10.1118/1.3058481 19291994

[45] Hussein M , Clark CH , Nisbet A . Challenges in calculation of the gamma index in radiotherapy ‐ Towards good practice. Phys Medica PM Int J Devoted Appl Phys Med Biol Off J Ital Assoc Biomed Phys AIFB. 2017;36:1‐11. doi:10.1016/j.ejmp.2017.03.001 28410677

[46] Celi S , Costa E , Wessels C , Mazal A , Fourquet A , Francois P . EPID based in vivo dosimetry system: clinical experience and results. J Appl Clin Med Phys. 2016;17(3):262‐276. doi:10.1120/jacmp.v17i3.6070 PMC5690938 27167283

[47] Oh KS , Jung K . GPU implementation of neural networks. Pattern Recognit. 2004;37(6):1311‐1314. doi:10.1016/j.patcog.2004.01.013

[48] Kirkby C , Sloboda R . Consequences of the spectral response of an a‐Si EPID and implications for dosimetric calibration. Med Phys. 2005;32(8):2649‐2658. doi:10.1118/1.1984335 16193795

[49] McNiven AL , Sharpe MB , Purdie TG . A new metric for assessing IMRT modulation complexity and plan deliverability. Med Phys. 2010;37(2):505‐515. doi:10.1118/1.3276775 20229859

